# Socioeconomic inequalities in non- coverage of full vaccination among children in Bangladesh: a comparative study of Demographic and Health Surveys, 2007 and 2017–18

**DOI:** 10.1186/s12889-022-12555-9

**Published:** 2022-01-27

**Authors:** Shobhit Srivastava, T. Muhammad, Rashmi Rashmi, Pradeep Kumar

**Affiliations:** International Institute for Population Sciences, Maharashtra 400088 Mumbai, India

**Keywords:** Vaccination, Children, Decomposition, Socioeconomic, India

## Abstract

**Background:**

Vaccination is considered as a powerful and cost-effective weapon against many communicable diseases. An increase in full vaccination among the most vulnerable populations in Bangladesh was observed in the last decade. This study aimed to capture the socioeconomic inequalities in non-coverage of full vaccination among children aged 12–23 months using the nationally representative data from the Bangladesh Demographic and Health Surveys (BDHS).

**Methods:**

Data for this study have been drawn from the 2007 and 2017–18 BDHS, which covered 10,996 and 20,127 ever-married women aged 15–49 years in 2007 and 2017–18, respectively. Binary logistic regression analysis was performed to find the factors associated with children who did not receive full vaccination. Further, the concentration index was used to observe the socioeconomic inequality for the outcome variable.

**Results:**

The proportion of children who did not get fully vaccinated decreased by more than 6 points (18.2 percent to 11.8 percent) between the years 2007 and 2017–18. In 2017–18, the odds of children who were not fully vaccinated were 58 percent and 53 percent less among mothers who had primary education in 2007 [adjusted odds ratio (AOR): 0.42; confidence interval (CI): 0.24–0.73] and 2017–18 [AOR: 0.47; CI: 0.23–0.94] respectively, compared to mothers with no education. The inequality for children who were not fully vaccinated had declined between two survey periods [concentration index (CCI) value of − 0.13 in 2007 and -0.08 in 2017–18]. The concentration of inequality in children with higher parity who did not receive full vaccination had increased from 5 percent in 2007 to 16.9 percent in 2017–18. There was a drastic increase in the socioeconomic inequality contributed by place of delivery from 2.9 percent (2007) to 60.5 percent (2017–18) among children who did not receive full vaccination.

**Conclusions:**

The present study provide eminent evidence that non-coverage of full vaccination is more prevalent among children from poor households in Bangladesh, which is mainly associated with factors like mother’s education, father’s education and working status and household wealth index across the two rounds. These factors suggest multifaceted pro-poor interventions that will protect them from hardship and reduce their socioeconomic inequalities in coverage of full vaccination.

**Supplementary Information:**

The online version contains supplementary material available at 10.1186/s12889-022-12555-9.

## Background

While inequality between countries has been declining, achieving Sustainable Development Goals (SDGs) is a major obstacle in poor resource settings due to within-country inequality in health [1]. Substantial progress has been made globally in the reduction of child mortality as a Millennium Development Goal [2]. Various evidence has shown that vaccination coverage at the recommended ages and intervals ensures that children are adequately protected from target diseases at all-times such as encephalitis [3]. Importantly, vaccination is considered as a powerful and cost-effective weapon against many of communicable diseases. Although the utmost priority was given to developing countries because of the higher prevalence of diseases and inadequate service delivery for immunisation, most of the unvaccinated children live in these countries [4–6].

A growing body of literature suggests that the socioeconomic inequalities still remain a major barrier to childhood vaccination coverage in low- and middle-income countries [7, 8]. Studies showed an absolute inequality in childhood vaccination coverage which is advantageous for male and urban residing children in the south and southeast regions [9–11]. Factors that hinder achieving complete childhood vaccination coverage include inadequacy of health services, lack of parental education, poverty, and traditional beliefs in society [12–14]. Evidence suggests that parental education, work status and household economic status are the most influential factors in reducing childhood vaccination inequality in poor societies [15–17]. Studies based on the Demographic and Health Surveys (DHS) also showed that full immunization was associated with the child’s age, sex and mother’s education and wealth [18–20]. Additionally, children of mothers with lower parity and higher birth interval of a preceding child in multiple studies were found to be more likely to be vaccinated [21, 22].

An encouraging increase in full vaccination among the most vulnerable populations in Bangladesh was observed in the last decade [23]. However, despite years of health and medical advancement, children still contact infectious diseases and inequalities in vaccination coverage and uptake persist, and deaths from vaccine-preventable diseases remain high in some parts of the country [24]. An earlier study revealed that almost half of the deaths occurring among children from poor socioeconomic groups could be prevented through immunisation alone [22]. The epidemiological studies conducted in this setting show an increased burden of infectious diseases observed in the impoverished parts like urban slums and poor regions of the country [25]. Also, in another study, full coverage of childhood vaccination was found to be positively associated with urban residence and media exposure [22].

Facility-based delivery has substantially increased in Bangladesh in the last decades, and a window of opportunity has been opened to raise the vaccination coverage [26, 27]. Since evidence shows that an age-appropriate vaccination raises the probability of children’s school enrolment [27], a better understanding of the determinants of inequalities in vaccination coverage is crucial for health intervention strategies. Although a couple of studies have reported findings on childhood vaccination, few of them have generated evidence on the inequalities in vaccination coverage in low and middle-income countries [7, 17, 28]. This study aimed to capture the socioeconomic inequalities in non-coverage of full vaccination status among children aged 12–23 months using the nationally representative DHS datasets.

## Methods

### Data

The study used data from the 2007 Bangladesh Demographic and Health Survey (BDHS) and 2017–18 BDHS, which are nationally representative surveys conducted by the National Institute for Population Research and Training (NIPORT) of the Ministry of Health and Family Welfare [29, 30]. The 2007 BDHS survey used the sampling frame provided by the list of census enumeration areas (EAs) with population and household information from the 2001 Population Census. Bangladesh is divided into six administrative divisions. In turn, each division is divided into *zilas*, and each *zila* into *upazilas*. Rural areas in an *upazila* are divided into union *parishads* (UPs), and UPs are further divided into *mouzas*. Urban areas in an *upazila* are divided into wards, and wards are subdivided into *mahallas*. EAs from the census was used as the Primary Sampling Units (PSUs). The survey is based on a two-stage stratified sample of households. At the first stage of sampling, 361 PSUs were selected. The selection of PSUs was done independently for each stratum and with probability proportional to PSU size in terms of a number of households. The urban areas of each division were further subdivided into three strata: statistical metropolitan areas (SMAs), municipality areas, and other urban areas. In all, the sample consisted of 22 strata because Barisal and Sylhet do not have SMAs. The 361 PSUs selected in the first stage of sampling included 227 rural PSUs and 134 urban PSUs. On average, 30 households were selected from each PSU, using an equal probability systematic sampling technique. A total of 10,996 ever-married women aged 15–49 from 10,400 households were interviewed with a response rate of 98.5% and 99.5%, respectively.

The 2017–18 survey used a sampling frame from the list of enumeration areas (EAs) of the 2011 Population and Housing Census of the People’s Republic of Bangladesh, provided by the Bangladesh Bureau of Statistics (BBS). The primary sampling unit (PSU) of the survey is an EA with an average of about 120 households. The survey is based on a two-stage stratified sample of households. In the first stage, 675 EAs (250 in urban areas and 425 in rural areas) were selected with probability proportional to EA size. In the second stage of sampling, a systematic sample of an average of 30 households per EA was selected to provide statistically reliable estimates of key demographic and health variables for the country as a whole, for urban and rural areas separately [31]. A total 20,127 ever-married women aged 15–49 years from 19,457 households were interviewed with a response rate of 98.8% and 99.4%, respectively.

The BDHS obtained detailed information on fertility levels, marriage, fertility preferences, awareness and use of family planning methods, breastfeeding practices, nutritional status of women and young children, childhood mortality, maternal and child health, and knowledge and attitudes regarding HIV/AIDS and other sexually transmitted infections. The detailed information on the survey is given elsewhere [30]. The effective sample size for the current analysis was 1157 (2007) and 1660 (2017–18) women aged 15–49 years who had given birth at home or a health facility during three years preceding the survey.

### Variable description

#### Outcome variable

The outcome variable was whether the child received all basic vaccination or not. Full vaccination includes one dose of BCG against tuberculosis, three doses of DPT (diphtheria, whooping cough, and tetanus), three doses of oral polio vaccine and one dose of measles vaccine among 12–23 months children [32]. The variable was coded as 0 “received full vaccination” and 1 “did not receive full vaccination”. The DHS collected the full vaccination status of children from the two sources. Primarily immunisation record cards were provided by mothers, but if these were absent in the DHS the data collectors used mothers’ verbal reports of children’s immunisation status [33].

#### Equity stratifier

The wealth index was the equity stratifier in the present study. The wealth index has a natural ordering and known as an ordered stratifier used in several socioeconomic-related inequality studies and has a high predictive value in low and middle-income countries [34, 35]. It was coded as poorest, poorer, middle, richer, and richest [32, 34]. Households were given scores based on the number and kinds of consumer goods they own, ranging from a television to a bicycle or car, and housing characteristics such as the source of drinking water, toilet facilities, and flooring materials. These scores are derived using principal component analysis. National wealth quintiles are compiled by assigning the household score to each usual (de jure) household member, ranking each person in the household population by their score, and then dividing the distribution into five equal categories [32, 34].

#### Explanatory variable

The study added the explanatory variables based on the literature available [8, 23, 36]. The sex of the child was coded as male and female, the mothers’ age was coded as 15–19, 20–14, 25–29 and 30 + years, mothers’ and fathers’ educational status was coded as not educated, primary, secondary and higher. Mothers’ and Fathers’ working status was coded as not working and working. Media exposure which includes exposure to television, radio and newspaper, was coded as exposed to anyone and not exposed [37]. Parity was coded as 1, 2, 3 and 4 and above. Preceding childbirth interval was coded as less than 24 months, 24–36 months and more than 36 months [33, 38]. Antenatal care visits were coded as no visit, 1–3 visits and 4 or above visits [38]. Postnatal care within two days of delivery was coded as no and yes [33]. The place child delivered was coded as home and health facility.

Religion was coded as Islam and others, and residential status was coded as urban and rural. Administrative divisions were provided in the survey as Barisal, Chittagong, Dhaka, Khulna, Rajshahi and Sylhet. To be noted in Bangladesh Demographic and Health Survey 2017–18 had eight administrative divisions, namely Barisal, Chittagong, Dhaka, Khulna, Mymensingh Rajshahi, Rangpur and Sylhet. For analytical reasons, Mymensingh was merged in Dhaka and Rangpur was merged in Rajshahi as these were divided from these regions at certain points after 2004.

### Statistical analysis

Descriptive (percentage) along with bivariate analysis was used for carving out preliminary results. The Chi-square test was used to resemble the significance level (p-values) during bivariate association. Along with that, binary logistic regression analysis [39] was performed to estimate the association between outcome and explanatory variables.

### Concentration Index (CCI)

The concentration curve is obtained by plotting the cumulative proportion of outcome variables (vaccination status) on y-axis against the increasing percentage of the population ranked by the socioeconomic indicator (wealth index) on x-axis. The curves show that whether the socio-economic status related inequality in the outcome variable (on x-axis) prevails or not [32, 37]. If the curve is above the line of equality (45 degree line) that means the index value is negative; hence it shows that the outcome variable is disproportionally concentrated among the poor and vice-versa [32, 37]. Income-related inequality in the vaccination status was measured by the concentration index (CCI) and the concentration curve (CC), using the wealth score as the socioeconomic indicator and binary outcome as vaccination status [32, 37]. The concentration index is defined as twice the area between the concentration curve and the line of equality. The concentration index measures the inequality of one variable (vaccination status) over the distribution of another variable (wealth index) [40]. The index ranges from -1 to + 1, where the index value of 0 (zero) shows no socioeconomic inequality [40]. However, the positive value of the index shows pro-rich inequality and vice-versa. Additional on either scale higher the value, the higher the extent of socioeconomic inequality.

CCI (concentration index), WI (Wagstaff's index), and EI (Erreygers index) are all binary variables that condition the level of absolute inequality on the most unequal society, although their definitions of that state differ [41]. CCI responds to the issue of how far the society has progressed from a state in which the wealthiest individual owns all of the society's health units (without considering the upper bound of the variable) [41]. WI and EI, on the other hand, acknowledge the boundedness of the health variable; WI answers the question of how far the society is from a state where only the top half of the income distribution is healthy, regardless of prevalence, while EI answers the question of how far the society is from a state where only the top half of the income distribution is healthy, regardless of prevalence [41].

The study used Wagstaff decomposition analysis to decompose the concentration index. Wagstaff’s decomposition demonstrated that the concentration index could be decomposed into the contributions of each factor to the income-related inequalities [42]. For any linear regression model on health outcome (y) (vaccination status), such as.

1 $$y=\alpha +{\sum}_k{\beta}_k{x}_k+\varepsilon$$

The concentration index for y, C, can be written as follows,

2 $$C={\sum}_k\left({\beta}_k{\overline{x}}_k/\mu \right){C}_k+G{C}_{\varepsilon }/\mu$$

Where $\mu$ is the mean of y, ${\overline{x} }_{k}$ is the mean of ${x}_{k}$, ${C}_{k}$ is the concentration index for ${x}_{k}$ (defined analogously to C), and $G{C}_{\varepsilon }$ is the generalized concentration index for the error term ($\varepsilon )$. Equation (2) shows that C is equal to a weighted sum of the concentration indices of the k regressors, where the weight for ${x}_{k}$ is the elasticity of y with respect to ${x}_{k}$ $\left({\eta }_{k}= {\beta }_{k}\frac{{\overline{x} }_{k}}{\mu }\right)$. The residual component captured by the last term reflects the socioeconomic status related inequality in health that is not explained by systematic variation in the regressors by income, which should approach zero for a well-specified model [32]. Each contribution is the product of elasticity with the degree of economic inequality [32]. Moreover, the percentage contribution is obtained by dividing each absolute contribution by total absolute contribution multiplied by 100 to obtain the estimates [32, 38]. The positive contribution indicates the role of factors in the extent of higher inequality, and the negative contribution explains the extent of reduction in inequality. Multicollinearity was assessed using variance inflation factor (VIF) [43]. Svyset command was used in STATA 14 to account for complex survey design. Further, individual weights were used to make the estimates nationally representative. STATA 14 [44] was used to analyse the dataset.

## Results

### Socioeconomic and demographic profile of study population (Table 1)

**Table 1:** Socio-demographic profile of the study population in Bangladesh, 2007 and 2017–18

Background characteristics	2007		2017–18	
	**Sample**	**Percentage**	**Sample**	**Percentage**
**Sex of the child**				
Male	581	50.2	832	50.2
Female	576	49.8	828	49.9
**Mother's age (in years)**				
15–19	269	23.3	293	17.6
20–24	419	36.2	591	35.6
25–29	279	24.1	406	24.5
30 +	190	16.4	370	22.3
**Mother's educational status**				
Not educated	249	21.6	102	6.1
Primary	356	30.8	469	28.3
Secondary	472	40.8	787	47.4
Higher	80	6.9	302	18.2
**Mother's working status**				
Not working	914	79.0	1009	60.8
working	243	21.0	651	39.2
**Media exposure (Radio/television/newspaper)**				
Not exposed	439	37.9	545	32.8
Exposed to any one	718	62.1	1115	67.2
**Parity**				
1	416	36.0	601	36.2
2	309	26.7	563	33.9
3	196	17.0	294	17.7
4 +	236	20.4	203	12.2
**Preceding child interval (in months)**				
< 24 months	104	9.0	103	6.2
24–36 months	171	14.8	139	8.4
> 36 months	882	76.2	1418	85.4
**Ante-natal care visits**				
No visits	461	39.9	162	9.7
1–3 visits	422	36.4	741	44.6
4 + visits	274	23.7	758	45.7
**Post-natal care**				
No	767	66.3	860	51.8
Yes	390	33.7	800	48.2
**Place child was delivered**				
Home	927	80.2	823	49.6
Health facility	230	19.9	837	50.4
**Father's educational status**				
Not educated	357	30.8	239	14.4
Primary	342	29.6	582	35.1
Secondary	328	28.3	529	31.9
Higher	130	11.3	311	18.7
**Father's working status**				
Not working	33	2.9	31	1.9
working	1124	97.1	1629	98.1
**Wealth Index**				
Poorest	247	21.3	338	20.4
Poorer	229	19.8	344	20.7
Middle	237	20.4	318	19.2
Richer	223	19.3	329	19.8
Richest	221	19.1	330	19.9
**Religion**				
Islam	1063	91.9	1558	93.9
Others	94	8.1	102	6.1
**Residence**				
Urban	275	23.8	440	26.5
Rural	882	76.3	1220	73.5
**Region**				
Barisal	70	6.1	93	5.6
Chittagong	281	24.3	351	21.2
Dhaka	364	31.5	555	33.5
Khulna	91	7.9	142	8.6
Rajshahi	255	22.1	376	22.6
Sylhet	96	8.3	142	8.6
**Total**	1157	100.0	1660	100.0

Results showed that about half of the children were males in both the survey period (2007 and 2017–18). Further, a higher proportion (about 36 per cent) of mothers belonged to the age group of 20–24 years in both BDHS rounds. Moreover, the proportion of mothers with no education decreased drastically from 21.6 per cent in 2007 to 6.1 per cent in 2017–18, and the share of working mothers increased from 21 per cent to 39.2 per cent during the last one decade. Mass media exposure among mothers increased from 62.1 per cent in 2007 to 67.2 per cent in 2017–18. However, mothers with 4 + parity decreased from 20.4 per cent to 12.2 per cent during the last one decade. The proportion of mothers who visited 4 + times for antenatal care increased by 22 point and the proportion of mothers who received postnatal care increased by about 15 points during two survey periods. There was a drastic increase among mothers who gave birth in the health facility between 2007 to 2017–18 (19.9 per cent to 50.4 per cent).

### The proportion of children who did not receive full vaccination (12–23 months) in Bangladesh (Table 2)

**Table 2:** Percentage of children who did not receive full vaccination (12–23 months) in Bangladesh, 2007 and 2017–18

Background characteristics	2007		2017–18	
	**Percentage**	***p*** **-value**	**Percentage**	***p*** **-value**
**Sex of the child**		0.928		0.576
Male	18.8		12.5	
Female	17.5		11.0	
**Mother's age (in years)**		0.558		0.048
15–19	19.9		12.9	
20–24	16.8		14.0	
25–29	15.4		12.1	
30 +	22.7		6.8	
**Mother's educational status**		< 0.001		< 0.001
Not educated	28.2		19.4	
Primary	22.0		16.2	
Secondary	12.2		9.2	
Higher	4.8		8.9	
**Mother's working status**		0.146		0.100
Not working	17.6		12.4	
working	20.3		10.7	
**Media exposure (Radio/television/newspaper)**		< 0.001		0.036
Not exposed	23.0		13.7	
Exposed to anyone	15.2		10.8	
**Parity**		0.011		0.855
1	16.7		10.8	
2	13.2		11.9	
3	18.0		14.4	
4 +	27.2		10.5	
**Preceding child interval (in months)**		< 0.001		0.012
< 24 months	28.0		10.7	
24–36 months	20.7		20.4	
> 36 months	16.5		11.0	
**Ante-natal care visits**		< 0.001		< 0.001
No visits	25.0		17.2	
1–3 visits	17.1		14.3	
4 + visits	8.3		8.1	
**Post-natal care**		< 0.001		0.101
No	21.3		11.1	
Yes	11.9		12.4	
**Place child was delivered**		< 0.001		< 0.001
Home	20.5		14.2	
Health facility	8.7		9.4	
**Father's educational status**		< 0.001		0.009
Not educated	26.5		16.2	
Primary	15.4		12.5	
Secondary	17.0		11.3	
Higher	5.5		7.7	
**Father's working status**		0.173		0.229
Not working	22.6		20.1	
working	18.0		11.6	
**Wealth Index**		0.002		0.003
Poorest	20.1		13.4	
Poorer	24.6		14.3	
Middle	21.0		9.4	
Richer	12.9		13.1	
Richest	11.5		8.4	
**Religion**		0.850		0.476
Islam	18.2		11.8	
Others	17.5		10.7	
**Residence**		< 0.001		0.731
Urban	13.7		11.8	
Rural	19.5		11.7	
**Region**		< 0.001		0.049
Barisal	9.8		13.9	
Chittagong	22.7		13.4	
Dhaka	17.7		12.0	
Khulna	11.1		8.3	
Rajshahi	14.5		9.1	
Sylhet	29.2		15.7	
**Total**	18.2		11.8	

Note: *p*-value based on chi-2 test

It was found in Table 2 that the proportion of children who did not get fully vaccinated decreased by more than 6 points (18.2 (confidence interval (CI): 16.0–20.6) per cent to 11.8 per cent (CI: 10.4–13.6)) between 2007 and 2017–18. The percentage of children who did not receive full vaccination were higher among women with no education in both the rounds of survey (28.2 per cent in 2007 and 19.4 per cent in 2017–18). The prevalence of children who had not fully vaccinated declined among women who had no media exposure (23 per cent in 2007 and 13.7 per cent in 2017–18) or media exposure (15.2 per cent in 2007 and 10.8 per cent in 2017–18), though it was higher among an unexposed group of women. Women who did not receive antenatal care had a higher percentage of children who were not fully vaccinated in both the survey periods. Moreover, women who delivered at home had a higher percentage of children who were not fully vaccinated. Father’s education has a significant negative association with children who were not fully vaccinated. The prevalence of children who were not fully vaccinated was significantly higher in the Sylhet division of Bangladesh (29.2 per dent in 2007 and 15.7 per cent in 2017–18), though it declined during the last one decade.

Estimates from logistic regression analysis for children who did not receive full vaccination by background characteristics (2007 and 2017–18) are presented in Table 3. Pooled analysis estimates shows that the odds of not getting fully vaccine was significantly lower in 2017–18 in reference to 2007 [AOR: 0.60; CI: 0.49, 0.74]. In 2017–18, the likelihood of children who did not receive full vaccination were 46 per cent and 75 per cent less likely among mothers who belonged to 25–29 [AOR: 0.54; CI: 0.27–1.08] and 30 + years age group [AOR: 0.25; CI: 0.11–0.61], respectively compared to mothers who were in the age group of 15–19 years. With reference to uneducated mothers, the odds of children who were not fully vaccinated were 58 per cent and 53 per cent less among mothers who had primary education in 2007 [AOR: 0.42; CI: 0.24–0.73] and 2017–18 [AOR: 0.47; CI: 0.23–0.94] respectively. Similarly, in 2017–18, the likelihood of children who did not receive full vaccination was 42 per cent less among mothers who delivered at a health facility compared to those who delivered the baby at home [AOR: 0.58; CI: 0.32–0.98]. Lastly, through pooled estimates, it was found that the odds of not getting fully vaccinated was significantly lower in 2017–18 in reference to 2007 [AOR: 0.60; CI: 0.49, 0.74].

**Table 3:** Estimates for logistic regression analysis for children who did not receive full vaccination (12–23 months) by background characteristics in Bangladesh, 2007 and 2017–18

Background characteristics	2007	2017–18	Pooled estimates AOR 95% CI
	**AOR 95% CI**	**AOR 95% CI**	
**Year**			
2007			Ref
2017–18			0.60***(0.49, 0.74)
**Sex of the child**			
Male	Ref	Ref	
Female	1.02(0.75,1.41)	0.86(0.61,1.21)	
**Mother's age (in years)**			
15–19	Ref	Ref	
20–24	0.83(0.51,1.35)	0.84(0.50,1.43)	
25–29	0.62(0.32,1.20)	0.54*(0.27,1.08)	
30 +	0.60(0.28,1.31)	0.25***(0.11,0.61)	
**Mother's educational status**			
Not educated	Ref	Ref	
Primary	0.88(0.58,1.33)	0.81(0.43,1.53)	
Secondary	0.42***(0.24,0.73)	0.47***(0.23,0.94)	
Higher	0.33*(0.10,1.07)	0.62(0.24,1.60)	
**Mother's working status**			
Not working	Ref	Ref	
working	1.26(0.84,1.87)	0.89(0.61,1.29)	
**Media exposure (Radio/television/newspaper)**			
Not exposed	Ref	Ref	
Exposed to anyone	0.99(0.68,1.42)	1.06(0.70,1.62)	
**Parity**			
1	Ref	Ref	
2	0.68(0.4,1.16)	1.25(0.73,2.15)	
3	0.92(0.48,1.78)	2.12***(1.01,4.46)	
4 +	1.01(0.47,2.18)	1.57(0.62,3.98)	
**Preceding child interval (in months)**			
< 24 months	Ref	Ref	
24–36 months	0.64(0.35,1.15)	1.99(0.90,4.36)	
> 36 months	0.54***(0.31,0.93)	1.31(0.63,2.73)	
**Ante-natal care visits**			
No visits	Ref	Ref	
1–3 visits	0.72*(0.50,1.05)	0.96(0.56,1.63)	
4 + visits	0.58*(0.34,1.01)	0.63(0.35,1.13)	
**Post-natal care**			
No	Ref	Ref	
Yes	1.02(0.65,1.62)	0.63(0.36,1.10)	
**Place child was delivered**			
Home	Ref	Ref	
Health facility	0.91(0.49,1.69)	0.58***(0.32,0.98)	
**Father's educational status**			
Not educated	Ref	Ref	
Primary	0.75(0.49,1.13)	0.82(0.50,1.35)	
Secondary	0.98(0.61,1.57)	0.90(0.51,1.60)	
Higher	0.68(0.28,1.67)	0.80(0.35,1.83)	
**Father's working status**			
Not working	Ref	Ref	
working	0.44*(0.19,0.98)	0.60(0.23,1.61)	
**Wealth Index**			
Poorest	Ref	Ref	
Poorer	1.25(0.77,2.04)	1.34(0.81,2.23)	
Middle	1.33(0.80,2.21)	0.83(0.46,1.53)	
Richer	1.28(0.70,2.33)	1.35(0.72,2.55)	
Richest	1.20(0.61,2.39)	0.94(0.42,2.11)	
**Religion**			
Islam	Ref	Ref	
Others	0.88(0.48,1.61)	1.15(0.56,2.37)	
**Residence**			
Urban	Ref	Ref	
Rural	1.43***(1.05,2.15)	0.85(0.55,1.30)	
**Region**			
Barisal	Ref	Ref	
Chittagong	3.06****(1.57,5.96)	1.03(0.55,1.95)	
Dhaka	2.09***(1.04,4.21)	0.80(0.44,1.47)	
Khulna	1.75(0.76,4.03)	0.64(0.28,1.47)	
Rajshahi	1.63(0.78,3.41)	0.73(0.39,1.38)	
Sylhet	3.38***(1.68,6.77)	1.03(0.54,1.97)	

Note: *AOR* Adjusted Odds Ratio; *if *p* < 0.1 **if *p* < 0.05 and ***if *p* < 0.01; *Ref* Reference; *CI* Confidence Interval; pooled estimates were adjusted for all the background characteristics

### Socioeconomic-related inequality among children who were not fully vaccinated in Bangladesh

**Fig. 1:**
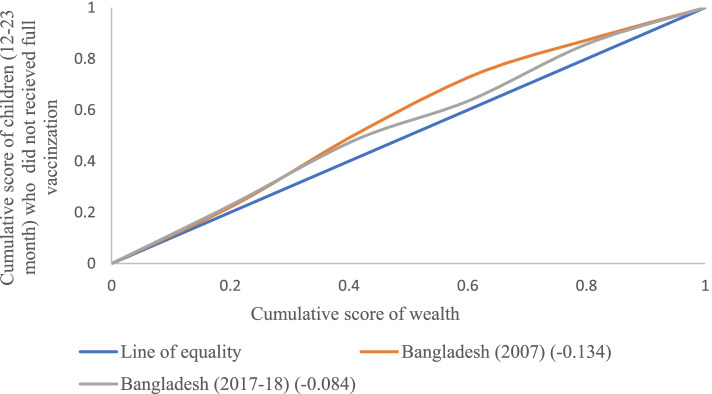
Concentration curve for children who were not fully vaccinated in Bangladesh during 2007 and 2017–18

Figure 1 presents the concentration curve for children who were not fully vaccinated in Bangladesh during 2007 and 2017–18. The inequality had declined between the two survey periods. For instance, Bangladesh witnessed a CCI value of − 0.13 in 2007 and -0.08 in 2017–18, which depicts that children who were not fully vaccinated was mainly concentrated among those from poor households. Moreover, Erreygers and Wagstaff normalized CCI are given in Table S1 (Additional file 1).

Table 4 represents the CI decomposition analysis results and depict how various socioeconomic and background factors of respondents contribute to the inequality among children who did not receive full vaccination in Bangladesh. The actual CCI was -0.134, whereas the calculated CCI was -0.023. The actual CCI represents the true concentration of outcome variable in higher of lower socio-economic strata; however, the calculated CCI reveals that how much does the over model explains out of total CCI. In this case, out of -0.134 (actual CCI) the model explained -0.023 (calculated CCI). Mother’s education explained 49.2 per cent (2007) and 38.4 per cent (2017–18) of socioeconomic status related inequality, whereas antenatal care explained 27.9 per cent (2007) and 40.3 per cent (2017–18) of socioeconomic status related inequality. Mother’s age was responsible for explaining 3.9 per cent and 13.2 per cent of socioeconomic status related inequality in 2007 and 2017–18, respectively. Moreover, parity explained 5% of socioeconomic related inequality in 2007, which increased to 16.9% in 2017–18. There was a drastic increase in the economic inequality contributed by place of delivery from 2.9 per cent (2007) to 60.5 per cent (2017–18) among children who did not receive full vaccine. The geographic region contributed 14.2 per cent and 3.8 per cent of the economic inequality (in poor) in the children who were not fully vaccinated in 2007 and 2017–18, respectively.

**Table 4:** Decomposition estimates for who did not receive full vaccination (12–23 months) by background characteristics in Bangladesh, 2007 and 2017–18

Background characteristics	2007					2017–18				
	**Elasticity**	**Concentration Index**	**Absolute contribution**	**% contribution**		**Elasticity**	**Concentration Index**	**Absolute contribution**	**% contribution**	
**Sex of the child**										
Male					-0.5					-0.3
Female	-0.006	-0.017	0.000	-0.5		-0.007	-0.004	0.000	-0.3	
**Mother’s age (in years)**										
15–19					3.9					13.2
20–24	-0.008	0.018	0.000	0.6		-0.006	0.044	0.000	2.8	
25–29	-0.017	0.056	-0.001	4.1		-0.015	-0.039	0.001	-6.1	
30 +	-0.009	-0.020	0.000	-0.7		-0.027	0.058	-0.002	16.5	
**Mother’s educational status**										
Not educated					49.2					38.4
Primary	-0.012	-0.190	0.002	-9.6		-0.008	-0.280	0.002	-22.1	
Secondary	-0.044	0.199	-0.009	37.1		-0.041	0.025	-0.001	10.8	
Higher	-0.008	0.657	-0.005	21.6		-0.011	0.456	-0.005	49.7	
**Mother’s working status**										
Not working					1.9					-7.8
working	0.004	-0.103	0.000	1.9		-0.005	-0.157	0.001	-7.8	
**Media exposure (Radio/television/newspaper)**										
Not exposed					5.9					-5.7
Exposed to one	-0.007	0.213	-0.001	5.9		0.003	0.190	0.001	-5.7	
**Parity**										
1					5.0					16.9
2	-0.016	0.004	0.000	0.3		0.010	0.036	0.000	-3.5	
3	-0.001	-0.027	0.000	-0.1		0.014	-0.076	-0.001	10.7	
4 +	0.005	-0.231	-0.001	4.8		0.005	-0.203	-0.001	9.7	
**Preceding child interval (in months)**										
< 24 months					3.4					2.7
24–36 months	-0.012	-0.234	0.003	-11.8		0.007	-0.101	-0.001	7.0	
> 36 months	-0.068	0.052	-0.004	15.2		0.023	0.018	0.000	-4.3	
**Ante-natal care visits**										
No visits					29.4					40.3
1–3 visits	-0.017	0.021	0.000	1.5		-0.005	-0.130	0.001	-6.3	
4 + visits	-0.019	0.347	-0.007	27.9		-0.023	0.198	-0.004	46.6	
**Post-natal care**										
No					0.7					-45.2
Yes	-0.001	0.318	0.000	0.7		-0.023	-0.190	0.004	-45.2	
**Place child was delivered**										
Home					2.9					60.5
Health facility	-0.001	0.487	-0.001	2.9		-0.027	0.215	-0.006	60.5	
**Father's educational status**										
Not educated					7.5					5.4
Primary	-0.021	-0.103	0.002	-9.4		-0.008	-0.225	0.002	-18.0	
Secondary	-0.004	0.245	-0.001	4.4		-0.005	0.141	-0.001	6.6	
Higher	-0.005	0.575	-0.003	12.5		-0.004	0.450	-0.002	16.8	
**Father's working status**										
Not working					-2.1					-0.2
working	-0.071	-0.007	0.000	-2.1		-0.057	0.000	0.000	-0.2	
**Wealth Index**										
Poorest					0.3					4.5
Poorer	0.013	-0.376	-0.005	21.1		0.006	-0.385	-0.002	25.2	
Middle	0.009	0.027	0.000	-1.0		-0.002	0.014	0.000	0.3	
Richer	0.002	0.425	0.001	-3.1		0.006	0.404	0.003	-26.0	
Richest	0.005	0.809	0.004	-16.6		-0.001	0.801	0.000	5.0	
**Religion**										
Islam					-0.1					1.2
Others	0.001	0.048	0.000	-0.1		0.001	-0.139	0.000	1.2	
**Residence**										
Urban					6.7					-19.9
Rural	0.011	-0.147	-0.002	6.7		-0.014	-0.142	0.002	-19.9	
**Region**										
Barisal					-14.2					-3.8
Chittagong	0.032	0.116	0.004	-15.7		0.000	0.111	0.000	-0.5	
Dhaka	0.026	0.065	0.002	-7.1		-0.008	0.141	-0.001	11.2	
Khulna	0.004	0.019	0.000	-0.3		-0.004	-0.027	0.000	-1.0	
Rajshahi	0.011	-0.143	-0.002	7.0		-0.007	-0.197	0.001	-13.9	
Sylhet	0.014	-0.033	0.000	1.9		0.000	-0.111	0.000	0.3	
Total				100.0						100
Calculated CCI			-0.023					-0.010		
Actual CCI			-0.134					-0.084		
Residual			-0.110					-0.074		

*CCI* Concentration Index

## Discussion

Although the child vaccination program is considered one of the most cost-effective interventions worldwide, the present study provides strong evidence of socioeconomic gradient in non-coverage of full vaccination among Bangladeshi children. Using two rounds of BDHS, our study revealed that most of the non-coverage in full vaccination occurs in poor households over time (from 2007 to 2017–18). However, the decrement in the concentration of inequality from -0.134 in 2007 to -0.084 in 2017–18 among children, together with the pooled analysis results on non-coverage of full vaccination, suggested a reduction in inequality over time. Even if the inequality has reduced, the question arises what are the factors which are still hindering the coverage of full vaccination among children aged 12–23 months in Bangladesh. Further, the present analysis tried to decompose the socioeconomic inequalities in non-coverage of full vaccination in children to examine the significant associated factors.

The present estimates revealed that mother’s age and parity, mother’s and father’s education, antenatal care, place of delivery, preceding childbirth interval, and wealth index had a consistent positive association in explaining the socioeconomic inequality in non-coverage of full vaccination in children across the decade (2007 to 2017–18). This means that if the covariates are equally distributed among rich and poor, the socioeconomic inequality in receiving basic vaccination will be less pro-rich (i.e., favouring richer household children). Factors such as the working status of the mother, having mass media exposure among women, postnatal care of the child, residing in a rural area were also positively associated with socioeconomic inequality in 2007; however, over time (till 2017–18) these factors helped in narrowing the poor-rich gap in the non-coverage of full vaccination among children.

Mother’s age and education play a significant role in childhood vaccination. This study explicitly found that non-coverage of full vaccination was higher among mothers of lower age groups and those with lesser education. These results are consistent with the previous studies, which have shown that the younger age group mothers usually lack the knowledge of providing basic health care to their children [45]. Higher maternal education also creates a big difference in the approach of their child care as compared to those who are illiterate. We found that both mother and father education plays a prominent role in child vaccination coverage in both rounds, and these results were consistent with the Ethiopian nation's evidence [46, 47]. The present study found that 4 + antenatal visits and child delivery at the health facility were associated with the socioeconomic inequality in vaccination coverage, which is consistent with a Lancet study [48]. The study shows that mothers accessibility to antenatal-care services and proper care at the time of delivery helps them seek information for their child's health and record the child's birth status by health professionals. This, in return, helps the professionals to track the health and nutrition of mother and child.

Although media exposure among women had helped uplift the health of their children and family, the dilemma is that most of the women in Bangladesh have not even access to basic media [49]. This brings the disadvantage of providing them basic knowledge of protecting their child through vaccination by mass media sources. Before the era of growing mass media networks, mothers were provided knowledge and awareness face-to-face and through direct consultation. However, with the passing years, a shift in communication has been noticed worldwide. Such shift may affect the direct consultancy of women to health professionals who still lag in receiving simple media information. The results of the present study had shown the positive association of mass media exposure on socioeconomic inequality in vaccine uptake in 2007, but this was not the case in 2017–18. This contradicts a study from Ethiopia where radio and television exposure had favoured the vaccination uptake [50]. This brought the importance of mass media exposure to build knowledge about health over time in Bangladesh.

The household wealth quintile was highly associated with inequality in non-coverage child full vaccination. However, it has also been noticed that children from poor wealth quintile households witnessed higher vaccination prevalence. This may be due to high poverty-centered programs to uplift their health and survival status [51]. However, the rural children remain more disadvantaged than their urban counterparts. This could be explained by the poor basic services and transportation facilities across rural areas, restricting the family from taking their child for vaccination. The lack of basic vaccination among rural children is consistent with few previous studies [23, 52]. The geographical location of Bangladesh is also found to be associated with the socioeconomic inequality in lack of complete child vaccination. Vast inequalities across different regions were noticed in 2007 when observing the situation of lack in child vaccination and these are consistent with a 2011 Bangladesh Demographic Health Survey study [53]. Over the decade, the vaccination coverage has increased across all the regions, leaving little socioeconomic inequality in inter-regional vaccination coverage; however, it persists.

Over the decades, the prevalence of receiving full childhood immunization has increased across Bangladesh. However, there still exists some room for slackness when we talk about the universalisation of this program. Ample evidence from Bangladesh has shown different factors affecting childhood vaccination. However, limited evidence had brought forward the presence of socioeconomic inequality in non-coverage of child full vaccination across a decade (2007 to 2017–18). Moreover, the associated factors of such socioeconomic inequality are still unexplored. The present study explored the overtime dynamic of full vaccination coverage among children aged 12–23 months in Bangladesh and the prominent factors associated with such socioeconomic inequality. However, the study backs with some limitations too. The cross-sectional nature of the data does not allow us to establish causality. Moreover, recall bias of vaccination status may exist when the children do not have their vaccination cards.

## Conclusion

Although a decrement in the number of children who were unable to be fully vaccinated was noticed over the decade, there still exists the need to provide knowledge and awareness about the protective effect of childhood vaccination due to socioeconomic inequalities. The present study shows pro-poor inequality in non-coverage of full vaccination among 12–23 months aged children in Bangladesh, which is mainly associated with factors like mother’s education, father’s education and working status and household wealth index across the two rounds. These factors suggest multifaceted pro-poor interventions to protect them from hardship and reduce their socioeconomic inequalities. The women’s utilization of health services during pregnancy and during or after delivery were found to be associated with the child vaccination coverage. This brings the need for coordinated policy responses at the household level, primarily focusing on providing all basic health care services to women right from the beginning. Also, there is a need for sensitization among both women and men through different modes in which face-to-face consultancy can also be provided in weaker sections of society., The changing status in inequalities (i.e., from positive to negative) across the two rounds due to mass media exposure, proper postnatal care, residing in rural areas shows the achievement of government policies focused in these areas and insists for wide interventions across all regions to benefit from such policies. Moreover, researchers should also bring knowledge to policymakers to improve such approaches over time.

## Supplementary Information

**Additional file 1:** **Table S1.** CCI for non-coverage of full immunization of children in Bangladesh

## Data Availability

The study utilizes a secondary source of data that is freely available in the public domain through, https://dhsprogram.com/data/dataset/Bangladesh_Standard-DHS_2017.cfm?flag=1

## Abbreviations

SDG: Sustainable Development Goals
BDHS: Bangladesh Demographic and Health Survey
NIPORT: National Institute for Population Research and Training
EAs: Enumeration Areas
BBS: Bangladesh Bureau of Statistics
PSU: Primary Sampling Unit
BCG: Bacille Calmette-Guerin
DPT: Diphtheria-Pertussis-Tetanus
CI: Confidence Interval
AOR: Adjusted Odds Ratio
CCI: Concentration Index
CC: Concentration Curve
