# Supplementary material for: Socioeconomic inequalities in non- coverage of full vaccination among children in Bangladesh: a comparative study of Demographic and Health Surveys, 2007 and 2017–18
Source: BMC Public Health. 2022 Jan 27;22:183. doi: 10.1186/s12889-022-12555-9 (PMC8793237; doi:10.1186/s12889-022-12555-9)
Supplement: Supplementary file 1 — Additional file 1: Table S1. CCI for non-coverage of full immunization of children in Bangladesh [file 12889_2022_12555_MOESM1_ESM.docx]

| **Table S1**. CCI for non-coverage of full immunization of children in Bangladesh | | | | |
| --- | --- | --- | --- | --- |
| **Types of CCI** | **2007** | | **2017** | |
|  | **CCI** | **p-value** | **CCI** | **p-value** |
| Generalized CCI | -0.13 | <0.0001 | -0.08 | <0.0001 |
| Erreygers normalized CCI | -0.0485 | 0.0002 | -0.0197 | 0.0307 |
| Wagstaff normalized CCI | -0.05 | 0.0002 | -0.02 | 0.03 |
| CCI: Concentration Index |  |  |  |  |
